# Effects of Additives on Fermentation Quality, Nutritional Quality, and Microbial Diversity of *Leymus chinensis* Silage

**DOI:** 10.3390/microorganisms14010027

**Published:** 2025-12-22

**Authors:** Mingga Qi, Zhijun Wang, Yushan Jia, Gentu Ge

**Affiliations:** 1 Key Laboratory of Forage Cultivation, Processing and High Efficient Utilization, Ministry of Agriculture, Inner Mongolia Agricultural University, Hohhot 010019, China; 2 Key Laboratory of Grassland Resources, Ministry of Education, Inner Mongolia Agricultural University, Hohhot 010019, China; 3 College of Grassland Science, Resources and Environment, Inner Mongolia Agricultural University, Hohhot 010019, China

**Keywords:** microorganisms characteristics, fermentation, *Leymus chinensis*, nutritional composition

## Abstract

This study evaluated how different additives—*Lactiplantibacillus plantarum* (LP), *Lentilactobacillus buchneri* (LB), and a composite enzyme (CE)—affect the fermentation quality, nutritional value, and microbial community of *Leymus chinensis* silage. Fresh forage was wilted to 65% moisture, treated with additives (dissolved in distilled water), and vacuum-sealed in polyethylene bags for 60 days of ensiling. Fermentation parameters and nutritional composition were analyzed using standard methods (e.g., HPLC for organic acids, Kjeldahl for crude protein), and the microbial community was profiled via Illumina MiSeq sequencing of the 16S rRNA gene V3-V4 region. Data were subjected to one-way ANOVA and Duncan’s test in SAS. All additives significantly improved key fermentation parameters (*p* < 0.05). The LP treatment yielded the most favorable profile, with the lowest pH (4.26) and the highest lactic acid (6.52 g/kg DM) and acetic acid (2.58 g/kg DM) contents. LP also best preserved nutrients, showing the highest dry matter (581.62 g/kg FW), water-soluble carbohydrates (24.76% g/kg DM), and crude protein (7.09% DM) (*p* < 0.05). The CE treatment most effectively degraded fiber, resulting in the lowest acid detergent fiber (428.87% g/kg DM) and neutral detergent fiber (628.43% g/kg DM) (*p* < 0.05). Additives significantly reduced bacterial alpha-diversity but enriched beneficial phylum such as Bacillota and genus such as *Lentilactobacillus* spp. LB), while suppressing harmful genera. Correlation analysis confirmed LP was positively correlated with lactic acid and water-soluble carbohydrates (*p* < 0.0001). In conclusion, additives, particularly LP, enhance silage quality by modulating the microbial community.

## 1. Introduction

*Leymus chinensis* is a dominant species in the meadow steppes and arid steppes of the eastern Eurasian Steppe [1]. It plays an important role in ecological protection and restoration. Not only does it help conserve soil and water and prevent wind erosion and sand fixation, but it is also rich in various nutrients and possesses characteristics such as high yield, drought resistance, tolerance to poor soil conditions, and resistance to grazing [2,3]. As a kind of excellent forage resource [4], sheep grass can be used not only as fresh forage for direct feeding, but also processed into hay, silage and meal, providing long-term storage and utilization options for livestock producers [5,6]. Under the principle of “limited quantities + complementary feeding”, it can also be used for hindgut fermentative monogastric animals such as rabbits and horses [7]. With the rapid development of the sheep grass industry, its yield has increased year by year. However, due to a lack of supporting processing technologies, the quality and utilization rate of its grass products are generally low, leading to a waste of sheep grass resources [8]. When sheep grass is processed into hay, the drying process is slow and susceptible to rainfall and dew, which can cause the hay to mold and spoil. Inadequate drying can also result in hay with excessively high moisture content, making it prone to rot and deterioration [9]. Ensiling can effectively reduce the loss of dry matter and nutrients in forage, extend the supply period, maintain its original nutritional value and flavor, thereby improving palatability and utilization rate [10].

Silage is a complex process of microbial dynamics, mainly through lactic acid bacteria converting water-soluble carbohydrates (WSCs) into lactic acid and acetic acid, lowering the pH value, inhibiting the reproduction of harmful microorganisms, thereby stabilizing the fermentation quality of silage [11]. Silage fermentation quality is a critical determinant of its effective utilization by livestock, as it directly influences feed preservation, nutritional value, and ultimately, animal health and performance [12]. The primary indicators for evaluating silage fermentation quality predominantly include pH, the content of organic acids (such as lactic acid, acetic acid, propionic acid, and butyric acid), and ammonia-nitrogen [13]. The evaluation of nutritional quality in silage is also a crucial aspect, as it is directly related to animal health, growth and development, and production efficiency [12]. Furthermore, ensiling is driven by a complex succession of microbial populations, whose composition and dominance shift throughout the fermentation phases. In a study on alfalfa, it was found that lactic acid bacteria effectively suppress harmful microorganisms, thereby promoting the formation of high-quality silage [14]. The subsequent accumulation of lactic acid effectively suppresses the proliferation of spoilage organisms, leading to the optimal preservation of nutritional components [15].

LP, as the most common homofermentative lactic acid bacterium [16,17], can rapidly and efficiently convert water-soluble carbohydrates (WSCs) in raw materials into large amounts of lactic acid [18]. This quickly lowers the pH value of the silage environment, creating an acidic environment that is unfavorable for the survival of most spoilage bacteria and pathogens (such as *Clostridium*, Escherichia coli, yeast, and *mold*) [16,19]. LB as a typical heterofermentative lactic acid bacterium, can significantly improve the aerobic stability of silage feed, reduce the risk of mycotoxins, and enhance fiber digestibility [20,21]. Composite enzyme preparations typically contain cellulase, xylanase, β-glucanase, etc., which precisely degrade plant cell walls to effectively improve fermentation quality and enhance the nutritional value and digestibility of feed [22]. The aim of this study is to address a critical agricultural challenge: the disruption of hay production for sheep grass (sheepgrass) due to unfavorable weather conditions, which threatens the stable supply of this high-quality forage prioritized by ruminant livestock. Sheep grass is not only underrepresented in silage research but also inherently difficult to ensile—primarily due to its low abundance of natural symbiotic lactic acid bacteria and high fiber content, which often lead to poor fermentation outcomes. To fill this gap, we systematically assessed the effects of three specific additives (i.e., LP, LB, and a composite enzyme preparation) on sheep grass silage. Our goal is to identify the optimal additive scheme that enhances not only its fermentation quality (e.g., pH reduction, organic acid profile) and nutritional value (e.g., crude protein retention, fiber degradation) but also its microbial community structure (as reported in the main text’s microbial diversity analysis), thereby providing a viable alternative to hay and supporting the efficient utilization of sheep grass resources in animal husbandry.

## 2. Materials and Methods

### 2.1. Silage Preparation

The experiment was conducted in 2024 at the Science and Technology Park of Inner Mongolia Agricultural University, located in Hailiutu Village, Tumetzuo Banner, Hohhot City, Inner Mongolia Autonomous Region (40°31′17″ N, 111°23′46″ E). Sheep grass was harvested using a hand-held scythe on 24 June 2024 (note: the original “2025” was adjusted to align with the 2024 experiment timeline) during its milk stage. The harvested sheep grass was severed into 1–2 cm segments using a cutting machine (Model: 9Z-0.8, Zhengzhou Huazheng Machinery Co., Ltd., Zhengzhou, China) and desiccated to a 65% moisture content for silage production. The desiccation process was conducted at the Key Laboratory of the Ministry of Agriculture and Rural Affairs: samples were first dried in an oven (Model: DHG-9070A, Shanghai Jinghong Laboratory Instrument Co., Ltd., Shanghai, China) at 105 °C for 30 min, followed by drying at 65 °C until a constant weight was achieved.

The treatments consisted of: (i) *L. plantarum* (LP, 2 × 10^11^ CFU/g fresh weight, sourced from Inner Mongolia Hemei Kesheng Biotechnology Co., Ltd., Hohhot, China); (ii) *L*. *buchneri* (LB, 2 × 10^11^ CFU/g, sourced from Inner Mongolia Hemei Kesheng Biotechnology Co., Ltd., Hohhot, China); (iii) composite enzyme preparation (CE), which was composed of the following enzymes: xylanase (≥18,000 U/g), cellulase (≥50 U/g), β-mannanase (≥1800 U/g), β-glucanase (≥200 U/g), pectinase (≥200 U/g), medium-temperature α-amylase (≥1500 U/g), and neutral protease (1000 U/g); this composite enzyme preparation was sourced from Shandong Mycobacterium Longum Biotechnology, China. The enzyme activity units of the composite enzyme were measured at pH 6.0 and 37 °C using the colorimetric method recommended by the ISO with a microplate reader (Model: Multiskan GO, Thermo Fisher Scientific Inc., Waltham, MA, USA); (iv) no-addition control (CK); and (v) native pasture sample of sheep grass (ORI).

The additives were dissolved in 20 mL of distilled water and uniformly sprayed onto 600 g of material. A total of 600 g of the mixed feed was thoroughly combined and sealed in polyethylene bags (30 cm × 40 cm) sourced from Shijiazhuang Youlang Trading Co., Ltd., Shijiazhuang, China. These bags were subsequently vacuum-sealed using a vacuum extraction machine (Model: DZ400/2D, Wenzhou Dafeng Machinery Co., Ltd., Wenzhou, China). A total of 12 bags (4 treatments × 3 replicates) were maintained at ambient temperature (17–25 °C). Samples were collected for the determination of fermentation characteristics and chemical composition. Among these, the 60-day samples—where fermentation had stabilized—were chosen for microbial community analysis and functional prediction analysis.

### 2.2. Nutritional Composition and Fermentation Characteristics Analyses

Nutritional and fermentation characteristics were assessed at 0, 7, 15, 30, and 60 days. The dry matter (DM) content of the fresh and ensiled material samples was determined following oven drying at 65 °C for 48 h [22]. The crude protein (CP) level was assessed using the Kjeldahl method with a Kjeldahl nitrogen analyzer (Model: Kjeltec 8400, FOSS Analytical AB, Höganäs, Sweden) [23]. Neutral detergent fiber (NDF) and acid detergent fiber (ADF) were determined using an ANKOM A200i fiber analyzer (ANKOM Technology Corp, Fairport, NY, USA), with hemicellulose content computed as the difference between NDF and ADF. The water-soluble carbohydrate (WSC) content was assessed utilizing Thomas’ method [24] with a spectrophotometer (Model: UV-2550, Shimadzu Corporation, Kyoto, Japan).

Each sample (10 g) was weighed using an electronic balance (Model: ME204E, Mettler Toledo Instruments Co., Ltd., Shanghai, China) and combined with 90 mL of distilled water. The mixture was homogenized using a homogenizer (Model: LC-11L, Shanghai Jingxin Industrial Development Co., Ltd., Shanghai, China) for 2 min, then filtered to obtain the liquid extract using a vacuum filtration device (Model: SHZ-D(III), Henan Yuhua Instrument Co., Ltd., Zhengzhou, China). The pH was assessed using an acidity meter (Model: LEICI PHS-3C, Shanghai Yitian Scientific Instrument Co., Ltd., Shanghai, China). The content of lactic acid (LA), acetic acid (AA), propionic acid (PA), and butyric acid (BA) was quantified via high-performance liquid chromatography (HPLC, Model: Waters e2695, Waters Corporation, Milford, MA, USA) equipped with a UV detector. Separation was achieved on a reversed-phase C18 column (e.g., Agilent ZORBAX SB-Aq, Santa Clara, CA, USA, 4.6 × 250 mm, 5 μm or equivalent) maintained at 30 °C. The mobile phase consisted of 20 mM potassium dihydrogen phosphate buffer (pH 2.5) and acetonitrile at a volume ratio of 98:2, which was delivered isocratically at a flow rate of 0.8 mL/min. The detection wavelength was set at 210 nm, and the injection volume was 10 μL. Additionally, the content of ammonia nitrogen (NH_3_-N) was determined via phenol-hypochlorous acid colorimetric analysis using a microplate reader [25].

For components such as lactic acid, organic acids, crude protein, and fiber, we report on a dry matter basis (g/kg DM). This is crucial because it eliminates variations in moisture content between different treatment groups, allowing all nutrient concentrations to be compared on the same benchmark. This accurately reflects their nutritional density and fermentation efficiency; For ammonium nitrogen, due to its volatility, we routinely measure it immediately in fresh samples or extracts. Therefore, results are reported on a fresh weight basis (% FW or g/kg FW) to accurately reflect its actual content within the silage.

### 2.3. Sequencing and Analysis of Microbial Diversity

To analyze the microbial community composition, relative abundance, and diversity after 60 days of sheep grass silage fermentation, total microbial DNA was extracted from the silage samples. This analysis relied on 16S rRNA gene sequencing, a widely used approach for microbial profiling: the 16S rRNA gene, a segment of approximately 1500 bp from the bacterial small ribosomal subunit (30S), contains 10 conserved regions and 9 hypervariable regions (V1–V9). Conserved regions enable the design of universal primers, while hypervariable regions carry species-specific genetic signatures, making them ideal for taxonomic identification.

In this study, the hypervariable V3–V4 region of the 16S rRNA gene was selected for amplification by PCR using the primers 338F (5′-ACTCCTACGGGAGGCAGCAG-3′) and 806R (5′-GGACTACHVGGGTWTCTAAT-3′). The resulting PCR products were separated on a 2% agarose gel and purified using the AxyPrep DNA Gel Extraction Kit (Axygen Biosciences, Union City, CA, USA). After elution in Tris-HCl and verification by 2% agarose gel electrophoresis, the products were quantified with the QuantiFluor™-ST system (Promega Corporation, Madison, WI, USA). Subsequently, the purified amplicons were used to construct PE 2 × 300 libraries following the Illumina MiSeq platform guidelines. Finally, paired-end sequencing (2 × 300 bp) was performed on the Illumina MiSeq PE300 platform (Majorbio Bio-Pharm Technology Co., Ltd., Shanghai, China), with subsequent data analysis involving alignment against reference databases (e.g., Silva, Greengenes, RDP) to determine microbial species composition, relative abundance, and diversity.

### 2.4. Statistical Analyses

The chemical composition and fermentation quality metrics of the silages after the fixed fermentation period were analyzed using a one-way analysis of variance (ANOVA) in SAS 9.4 (SAS Institute, Cary, NC). The statistical Model assessed the effect of the treatment as the sole fixed factor. When the ANOVA indicated a significant effect (*p* < 0.05), differences among treatment means were compared using Duncan’s multiple range test.

Data visualization “was performed” using Origin 2021 (OriginLab Corporation, Northampton, MA). Microbial community data “were processed and analyzed” through the Majorbio I-Sanger Cloud Platform (https://www.i-sanger.com, accessed on 8 July 2025), which included quality control, operational taxonomic unit (OTU) clustering, and taxonomic annotation based on the 16S rRNA gene sequences. Specifically, raw sequences were denoised, chimeras were removed, and ASVs (Amplicon Sequence Variants) were identified using DADA2, followed by taxonomic classification against reference databases (e.g., Silva). Diversity indices (e.g., Shannon, Chao1) and community composition were calculated to characterize microbial structures.

Analysis completed using the Meiji Cloud Platform (https://cloud.majorbio.com/): First, the platform’s automated workflow processed 16S sequencing data, including quality control, ASV generation, and species annotation against the Silva database. Microbial genera with relative abundances ≥0.5% were selected to eliminate noise; Subsequently, the “Correlation Heatmap” tool was employed to select the top 10 dominant genera and key quality indicators (nutritional quality, fermentation quality), calculating Spearman’s correlation coefficients (adapted to microbial abundance data) and controlling false positive rates via FDR correction (FDR < 0.05); Finally, generate clustered heatmaps to visually represent the positive/negative correlations and their strengths between microbial communities and quality indicators.

## 3. Results

### 3.1. Effects of Different Additives on Fermentation Quality of Sheep Grass

Table 1 shows that additives significantly influenced all indicators except BA and NH_3_-N (*p* < 0.05). NH_3_-N content across treatments ranged from 0.06 to 0.08 (g/kg DM). BA appears only in the CK and LB groups, and exists in LB in extremely small quantities. The pH levels in the LP and LB groups were significantly lower than in the other two groups (*p* < 0.05). This pronounced acidity is a direct consequence of the substantial LA accumulation. The LP group exhibited significantly higher LA and AA contents than other groups, while the CK group had significantly lower LA content (*p* < 0.05). The CE group had significantly lower AA content than all other groups but the highest PA content (*p* < 0.05).

### 3.2. Effects of Different Additives on Nutritional Components of Sheep Grass

As shown in Table 2, the application of different additives significantly influenced all measured nutritional components of sheep grass silage (*p* < 0.05). The fresh raw material (ORI group) exhibited the highest values for WSC, EE, ADF, and NDF (*p* < 0.05), and was statistically on par with the LP group for DM and CP content (sharing the same superscript letter ‘A’). Among the ensiled groups, the LP treatment resulted in the highest preservation levels for DM, CP, and WSC (*p* < 0.05). The LB treatment led to the highest EE content (*p* < 0.05), which was not significantly different from the ORI group. In contrast, the CE treatment yielded the lowest values for ADF and NDF (*p* < 0.05). The untreated control group (CK) consistently showed the lowest or among the lowest values for DM, WSC, CP, and EE (*p* < 0.05).

### 3.3. Effects of Different Additives on Microbial Diversity of Sheep Grass

**Figure 1:**
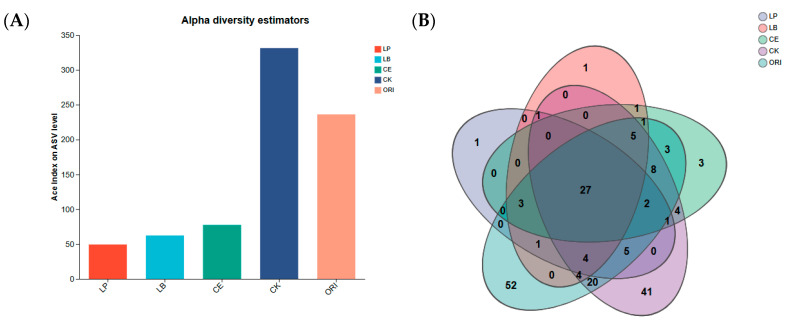
The community dissimilarities of sheep grass under different treatments (genus levels). (**A**) Alpha diversity estimatORIs of the silages’ bacterial community under various ensiling procedures; (**B**) Venn diagram showing distinct ORI similar bacterial OTUs in silages treated with various additives. LP *L*. *plantarum*, LB *L*. *buchneri*, CE composite enzyme, CK no-addition control, ORI native pasture sample for sheep grass.

Figure 1 clearly illustrates microbial diversity (at the genus level) across different treatments. Figure 1A reflects the magnitude of α-diversity indices, while Figure 1B analyzes the number of unique and shared species among groups. Figure 1A reveals that the α-diversity indices of the CK and ORI groups are notably high, reaching 331.33 and 236.16, respectively. In contrast, the indices of the additive groups are extremely low, with the LP group registering the lowest at 49.35, while the LB and CE groups recorded 62.39 and 77.42, respectively. Figure 1B reveals that 27 OTUs were shared across all groups. Among the additive groups, LP and LB each contained only 1 unique OTU, while CE had 3 unique OTUs. These numbers fall far short of the 41 and 52 unique OTUs found in CK and ORI, respectively. Although the above data focus solely on unique OTUs, they consistently indicate that microbial diversity was lower in the additive groups than in the non-additive controls (CK and ORI).

**Figure 2:**
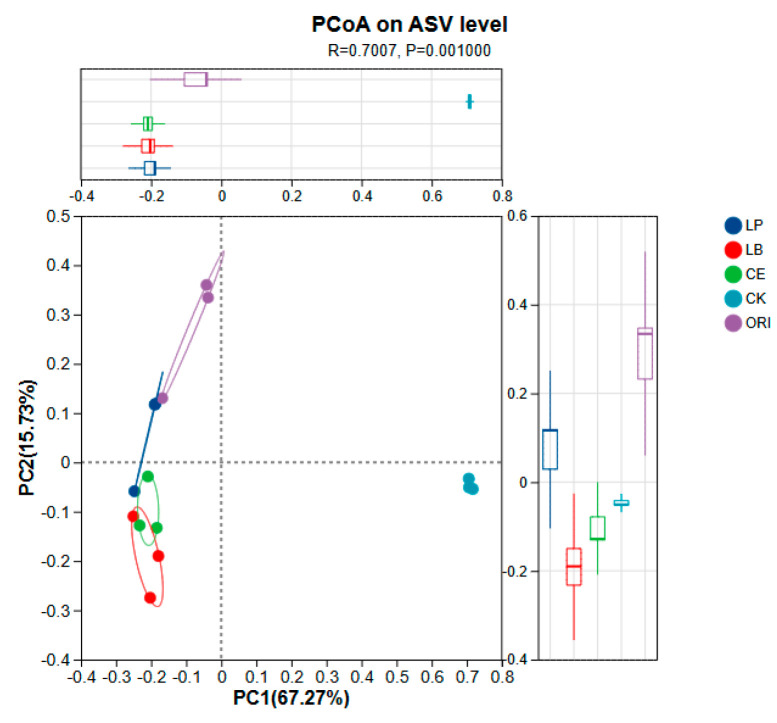
Principal CoORIdinate Analysis (PCoA) of the silages’ bacterial community under various ensiling procedures. LP *L. plantarum*, LB *L. buchneri*, CE composite enzyme, CK no-addition control, ORI native pasture sample for sheep grass.

Figure 2 illustrates the differences among sample groups. Principal Coordinate Analysis (PCoA) reveals significant clustering patterns between groups, with Principal Component 1 (PC1) and Principal Component 2 (PC2) explaining 67.27% and 15.73% of the total variance, respectively. The CK group exhibits extremely high reproducibility but shows substantial differences from other groups. Samples from the LB and CE groups demonstrate minor differences, while the LP group falls between the ORI and CE groups.

**Figure 3:**
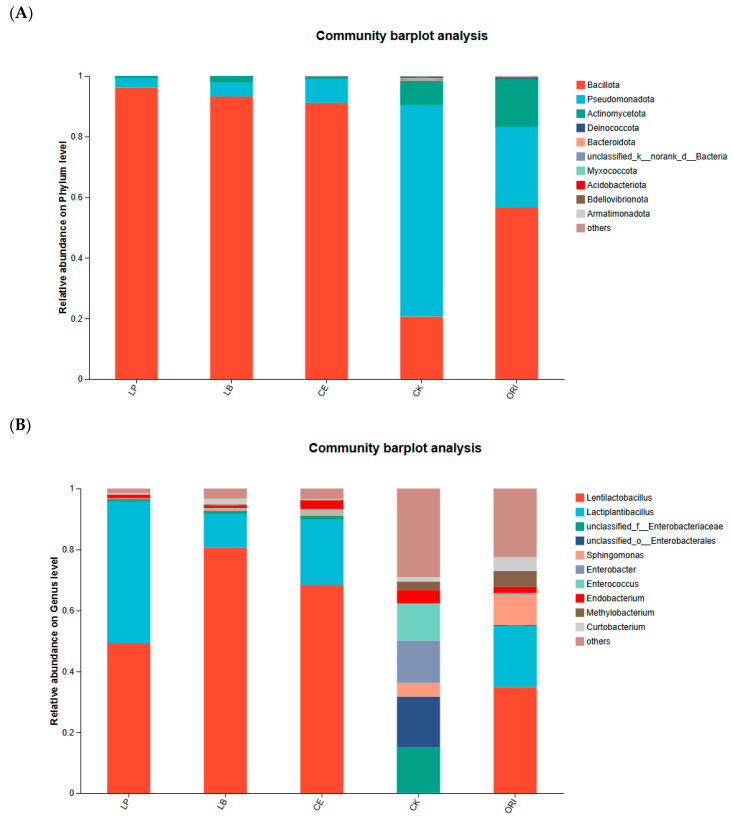
The taxonomic rank of bacterial communities (**A**) Relative abundance of bacterial community at phylum level; (**B**) relative abundance of bacterial community at genus level. LP *L. plantarum*, LB *L. buchneri*, CE composite enzyme, CK no-addition control, ORI native pasture sample for sheep grass.

In Figure 3, the top N most abundant species across all samples under each treatment are shown, along with the proportion distribution of different species. At the phylum level (Figure 3A), Bacillota is the most abundant dominant phylum. Among the additive groups LP, LB, and CE, they exhibit absolute advantages with values as high as 0.96, 0.93, and 0.91, respectively. However, the ORI group accounts for only 0.57, while the remaining portions are dominated by Pseudomonadota and Actinomycetota. CE showed absolute dominance at 0.96, 0.93, and 0.91, respectively. However, in the ORI group, it accounted for only 0.57, while Pseudomonadota and Actinomycetota were significantly present in other parts. In the CK group, the most abundant species was Pseudomonadota, followed by Bacillota and Actinomycetota. Genus-level species composition is shown in Figure 3B. Overall, *Lentilactobacillus* spp. dominated, exhibiting absolute dominance under LB (0.81) and CE (0.68) treatments. Under LP treatment, *Lentilactobacillus* spp. and *Lactiplantibacillus* spp. exhibited comparable abundances, jointly dominating the fermentation process. The species composition in the control group (CK) and original sample group (ORI) was more complex, encompassing nearly all species within the top 10 abundances and clearly observable in the figure. While the ORI group shared similarities in composition with the additive groups, the relative abundances of individual species differed significantly.

**Figure 4:**
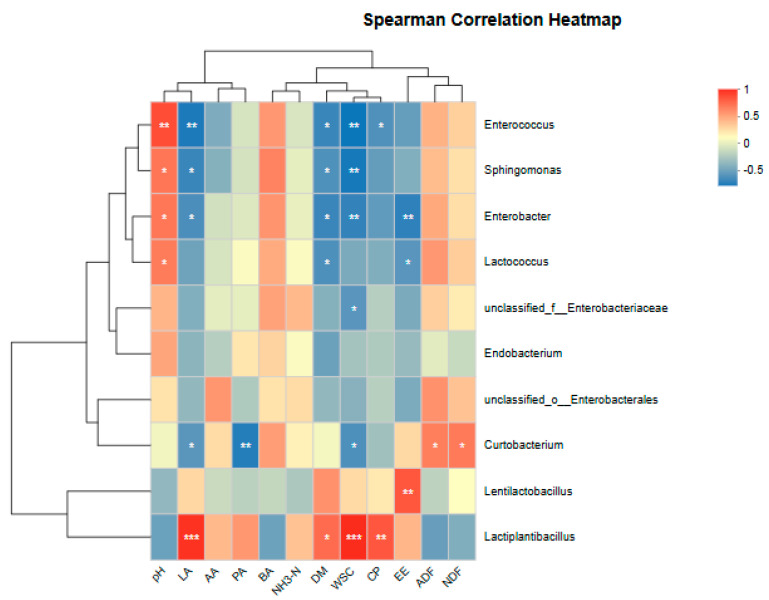
Effect of the end of silage fermentation on fermentation quality and chemical composition: genus level. (* *p* < 0.05; ** 0.001 < *p* < 0.01; *** *p* < 0.001). LP *L. plantarum*, LB *L. buchneri*, CE composite enzyme, CK no-addition control.

The correlation analysis between conventional nutritional and fermentation qualities and microbial composition is shown in Figure 4. pH exhibited significant positive correlations (*p* < 0.05) with *Enterococcus* spp., *Sphingomonas* spp., *Enterobacter* spp., and *Lactococcus* spp., while showing negative correlations with *Lentilactobacillus* spp. and *Lactiplantibacillus* spp. LA, DM, and WSC all showed significant negative correlations (*p* < 0.05) with *Enterococcus* spp., *Sphingomonas* spp., and *Enterobacter *spp. AA, CP, and EE also exhibited negative correlations with these three microorganisms. LA and WSC both showed extremely significant positive correlations (*p* < 0.0001) with *Lactiplantibacillus* spp., while simultaneously exhibiting significant negative correlations (*p* < 0.05) with *Curtobacterium* spp. DM and EE both showed significant negative correlations with *Lactococcus* spp. (*p* < 0.05). ADF and NDF were positively correlated with *Enterococcus* spp., *Sphingomonas* spp., *Enterobacter* spp., and *Lactococcus* spp., and significantly positively correlated with *Curtobacterium* spp. (*p* < 0.05), while negatively correlated with *Lentilactobacillus* spp. and *Lactiplantibacillus* spp.

## 4. Discussion

The fermentation quality of silage is collectively determined by key biochemical parameters, including pH, organic acid profiles, and NH_3_-N content [26,27]. As shown in Table 1, pH values differed significantly among groups (*p* = 0.0002): the CK group had the highest pH (4.72 ± 0.05), followed by the CE group (4.68 ± 0.03), while the LP and LB groups exhibited lower pH (4.26 ± 0.02 and 4.34 ± 0.05). This aligns with the lactic acid (LA) content trend—LA in the LP group reached 65.2 ± 0.42 g/kg DM, which was significantly higher than that in the CE (54.64 ± 1.93 g/kg DM), LB (49.59 ± 0.38 g/kg DM), and CK groups (41.90 ± 1.34 g/kg DM, *p* < 0.0001). As a homofermentative lactic acid bacterium, LP rapidly utilizes water-soluble carbohydrates (WSCs) under anaerobic conditions to produce large amounts of LA, thereby reducing pH [28,29]. For other organic acids: acetic acid (AA) content was highest in the LP group (25.81 ± 0.94 g/kg DM) (*p* = 0.0004), while the CE group had the lowest AA (12.33 ± 1.21 g/kg DM)—this may be related to the dominance of homofermentative bacteria promoted by CE, which prioritize LA production over AA, as a typical homofermentative pathway, this fermentation generates almost exclusively lactic acid, with only minimal acetic acid production [30,31]. Propionic acid (PA) showed the most obvious difference in the CE group (11.9 ± 0.04 g/kg DM) (*p* < 0.0001), significantly higher than the LP (7.23 ± 0.34 g/kg DM) and CK/LB groups (5.40 ± 0.29 and 4.77 ± 0.38 g/kg DM). This is likely because the composite enzyme decomposes cellulose and hemicellulose into fermentable sugars (e.g., xylose), which are used by Propionibacterium to synthesize PA via the succinate-propionate pathway [32]. Butyric acid is typically regarded as an indicator of abnormal fermentation or deterioration in silage, primarily produced by harmful microorganisms such as *Clostridium *spp. under anaerobic conditions [33]. Notably, butyric acid (BA) was only detected at a higher level in the CK group (1.72 ± 0.94 g/kg DM) (*p* = 0.0724, near significant), while the LP, LB, and CE groups had almost no BA (0.00 ± 0.00–0.11 ± 0.11 g/kg DM). Since BA is a marker of abnormal fermentation (produced by Clostridium [34]), the low BA in additive groups indicates effective inhibition of harmful microorganisms and better preservation of silage quality. Regarding NH_3_-N (an indicator of protein decomposition), no significant differences were observed among groups (*p* = 0.4972), with all groups maintaining low levels (0.63 ± 0.00–0.83 ± 0.00%FW). This suggests that all treatments, including CK, achieved good protein preservation, which may be related to the overall low pH of the silage (≤4.72) inhibiting proteolytic bacteria [35]. In summary, the LP and CE groups exhibited superior phenotypes in terms of fermentation quality.

The ensiling process aims not only to preserve forage but to enhance or maintain its nutritional value. This study demonstrates that additive treatments, particularly LP, LB, and CE, differentially improved the fermentation profile and, more importantly, led to distinct and valuable nutritional outcomes for sheep grass silage compared to the untreated control (CK). DM content, a critical factor in silage production, decreased post-fermentation, as expected [36,37]. Specifically, the CK and CE groups showed significant DM losses, with DM contents dropping to 494.73 ± 6.44 g/kg FW and 541.05 ± 7.13 g/kg FW, respectively, the LP treatment achieved the most effective preservation of overall nutritive value, as evidenced by its highest dry matter (DM) content (581.62 g/kg FW), which was statistically equivalent to the original fresh material (ORI). This is likely because the rapid acidification by LP suppressed the respiration and metabolism of aerobic spoilage microorganisms, thereby reducing DM losses-consistent with the lower DM loss observed in lactic acid bacteria-treated legume silage [38]. The ensiling process consumed a substantial amount of WSC, resulting in the ORI group retaining the highest WSC content (46.44 ± 0.29 g/kg DM), which was significantly higher than all treatment groups [39]. Notably, the LP group (70.94 ± 0.43 g/kg DM) exhibited a higher CP content than all other groups, including the ORI group. This phenomenon can be attributed to the rapid utilization of WSC by LP for lactic acid production, which led to a swift decline in environmental pH. The resulting acidic environment effectively suppressed the activity of plant-derived proteases, such as carboxypeptidase and serine protease, thereby attenuating the rate of protein degradation [40]. Consequently, the relative retention of crude protein (numerator) exceeded the loss in total dry matter (denominator), leading to an apparent increase in CP content—a “concentration effect” due to preferential dry matter loss. Similarly, the highest ether extract (EE) content observed in the LB group aligns with the mechanistic explanation above. The rapid establishment of lactic acid–dominant fermentation by LP inhibited the metabolic activity of spoilage microorganisms, thereby reducing overall dry matter losses (e.g., via CO_2_ respiration and leachate). As the most energy-dense component, fat underwent the least absolute degradation, and its proportion increased in the remaining dry matter, manifesting as an elevated EE content in the final silage [41]. Following the ensiling process, both ADF and NDF contents exhibited varying degrees of reduction compared to the ORI (ADF: 478.92 ± 3.40 g/kg DM; NDF: 704.82 ± 5.88 g/kg DM). This occurs because organic acids (lactic acid, acetic acid, etc.) produced during ensiling, in combination with plant/microbial enzymes, act synergistically to hydrolyze hemicellulose (the primary component of NDF) and cellulose (the primary component of ADF) through acid-enzyme combined hydrolysis. This process generates soluble sugars or organic acids, leading to a reduction in fiber content [42]. The CE group, containing cellulase and hemicellulase, achieved the greatest reduction: its ADF content (428.81 ± 4.16 g/kg DM) was 10.46% lower than the ORI, and its NDF content (628.43 ± 4.08 g/kg DM) was 10.84% lower than the ORI—far exceeding the reductions in LP (ADF: 438.53 ± 0.44 g/kg DM; NDF: 656.51 ± 2.73 g/kg DM) and CK groups.

Both alpha diversity analysis (including observed OTUs, Shannon, and Simpson indices) and Venn diagrams indicated that the CK and ORI groups maintained higher microbial diversity compared to the treated silages. This pattern can be attributed to the inhibitory effect of silage additives and their metabolites (e.g., acetic acid and phenylacetic acid) on aerobic bacteria, yeasts, and fungi, leading to a marked reduction in previously dominant taxa. In contrast, the CK and ORI groups, which were not subjected to such suppression, retained a richer and more even community structure, as reflected in their higher diversity indices [43,44]. PCoA further revealed that the microbial profile of the LP group closely resembled that of the ORI group. This similarity is likely due to the fact that LP is a natural epiphytic colonist of fresh forage. Upon inoculation, it rapidly occupied a dominant ecological niche, steering the community composition to converge toward the original epiphytic structure within a short timeframe. This phenomenon of microbial succession reverting toward the initial community state has also been documented in earlier studies [44,45,46].

This study found that Bacillota, Pseudomonadota, and Actinomycetota were present in all treatment groups at the conclusion of fermentation, with Bacillota being the predominant phylum [47,48]. The higher abundance of Pseudomonadota in the ORI and CK is indeed a common phenomenon and is typically regarded as an indicator of slow or failed fermentation. This is primarily due to the prolonged aerobic phase during the initial stage of silage fermentation and insufficient lactic acid fermentation, which leads to the proliferation of aerobic or facultative anaerobic Pseudomonadota [49]. This study observed a significant presence of *Lentilactobacillus* spp. in the treatment group supplemented with *Lactobacillus* spp., a bacterial group essential for the aerobic stability of silage [50]. The additive group and ORI group exhibited similar species composition but differing species proportions. This discrepancy arises because additives naturally occur on fresh forage surfaces at high abundance; inoculation merely rapidly amplifies their numbers, resulting in an early-stage community structure that is “replicated as-is” [8,51]. The additive group subsequently suppressed the invasion of exogenous contaminants, leading to a predominance of lactic acid bacteria. In contrast, the ORI group harbored a greater diversity of other contaminants. The CK group predominantly contained *unclassified_f*__Enterobacteriaceae, *unclassified_o*__Enterobacterales, *Enterobacter* spp., and *Enterococcus* spp. These are primarily spoilage indicator bacteria. Their proliferation consumes soluble sugars and produces undesirable metabolites such as ammonia and amines, thereby degrading silage quality [52,53,54].

Variations in microbial community diversity reflect corresponding shifts in fermentation characteristics. To systematically elucidate the interrelationships among chemical composition, fermentation parameters, and microbial assemblages, correlation analyses were conducted. *Enterococcus* spp., which thrive under alkaline conditions, is frequently associated with silage exhibiting high pH, indicative of suboptimal fermentation [55]. *Sphingomonas* spp. have been reported to occur at relatively high abundance in neutral to alkaline environments such as certain soils and water bodies [56]. As a facultative anaerobe, *Enterobacter* spp. often proliferate during the initial phase of ensiling or under insufficient anaerobic conditions, typically coinciding with a delayed pH drop or even a rise in pH, and is effectively suppressed in well-fermented, low-pH silage. In contrast, although *Lactococcus* spp. belong to the lactic acid bacteria, its growth optimum lies in the neutral pH range (approximately 6.3–6.9), and it is sensitive to strongly acidic conditions, which compromise its acid tolerance and restrict its proliferation under low-pH environments [57]. Therefore, pH showed a significant positive correlation with *Enterococcus* spp., *Sphingomonas* spp., *Enterobacter* spp., and *Lactococcus* spp. *Lactiplantibacillus* spp. and *Lentilactobacillus* spp. rapidly utilize WSC to produce large amounts of lactic acid, acting as the primary drivers behind the sharp decline in silage pH. This creates a positive feedback loop: lactic acid bacteria proliferation → acid production → pH decline → suppression of competitors → creation of a more favorable ecological niche for lactic acid bacteria → further proliferation of lactic acid bacteria. Consequently, lower environmental pH levels often correlate with higher abundances of these key lactic acid bacteria [58,59]. *Lactiplantibacillus* spp. can efficiently convert nearly all WSC (such as glucose and fructose) into lactic acid. Consequently, the greater the amount of available WSC in the environment, the stronger their growth and acid-producing capacity, exhibiting an extremely high positive correlation [40,60]. *Curtobacterium* spp. belong to the Actinomycetota. Many actinomycetes are better adapted to neutral or slightly alkaline environments and typically lack the highly efficient glycolysis and acid tolerance characteristic of lactic acid bacteria. The LA produced by *Lactiplantibacillus* spp. causes a sharp drop in pH, creating a strongly acidic environment. This environment represents a significant stress for many bacteria, including *Curtobacterium* spp., strongly inhibiting their growth and even causing death, thus exhibiting a negative correlation [61,62]. *Enterococcus* spp. thrive in alkaline environments and metabolizes cellulose/hemicellulose-derived oligosaccharides and cellobiose through heterofermentative pathways, yielding limited lactic acid that is insufficient to reduce ambient pH. Consequently, it predominates in high-fiber, low-acid silage conditions. *Enterobacter* spp., which favor neutral pH, secrete cellulases that degrade structural polysaccharides into fermentable sugars, while simultaneously producing ammonia and amines. These metabolic activities contribute to dry matter loss and a relative increase in fiber content [63]. *Sphingomonas* spp. participate directly in fiber degradation by excreting exo-cellulases and hemicellulases, leading to its enrichment in micro-niches with elevated ADF/NDF levels. Similarly, *Curtobacterium* spp. utilize plant cell wall components such as xylan and pectin, emerging as a dominant taxon under high-fiber, low-acidity regimes; its abundance fluctuates in concert with fiber content dynamics [64]. In contrast, *Lentilactobacillus* spp. and *Lactiplantibacillus* spp. act as pivotal drivers of silage fermentation, functioning as both initial colonizers and dominant acid producers. These taxa depend critically on WSC as substrates for rapid growth and copious lactic acid synthesis. Under WSC-depleted conditions, their proliferative and acidogenic capacities are compromised, diminishing their competitive edge within the microbial community. Hence, high fiber content—often concomitant with low WSC availability—fosters an ecological niche unfavorable to homofermentative lactic acid bacteria, accounting for their negative correlation with fiber-rich environments [2,65].

In terms of fermentation quality, LP acted as a direct and efficient driver, achieving the lowest pH (4.26) and highest lactic acid (65.2 g/kg DM), which effectively suppressed undesirable fermentation (0.0 g/kg DM butyric acid). In contrast, CE did not excel in rapid acidification but induced a unique metabolic shift, yielding the highest propionic acid (11.9 g/kg DM). Regarding nutritional outcomes, LP excelled in preservation. It maintained dry matter content (581.6 g/kg FW) equivalent to fresh forage and secured the highest crude protein (70.9 g/kg DM) among silages. Conversely, CE functioned as a substrate modifier, delivering the greatest reduction in fiber, with the lowest ADF (428.8 g/kg DM) and NDF (628.4 g/kg DM), indicating enhanced potential digestibility. The underlying microbial shifts towards simplified, LAB-dominated communities support these phenotypic results. In conclusion, LP optimizes preservation through rapid acidification, whereas CE enhances digestibility via fiber degradation. This comparison provides a clear rationale for selecting additives based on specific quality targets.

## 5. Conclusions

This study investigated the effects of *L*. *plantarum* (LP), *L*. *buchneri* (LB), and a composite enzyme (CE) on the fermentation quality, chemical composition, and bacterial community of sheep grass silage. The LP treatment yielded the most pronounced improvement, achieving the lowest pH (4.26) and the highest lactic acid content (65.24 g/kg DM), which were significantly superior to the control (CK). Consequently, the LP group also best preserved dry matter, water-soluble carbohydrates, and crude protein. The LB treatment effectively produced acetic acid and uniquely enhanced ether extract preservation. The CE treatment significantly reduced fiber content (ADF and NDF) and generated the highest propionic acid. All additives dramatically simplified the epiphytic bacterial community, shifting dominance to beneficial genera like *Lentilactobacillus* spp. and *Lactiplantibacillus* spp., which strongly correlated with improved fermentation parameters. In summary, the additives, particularly LP, significantly enhanced silage quality by driving an efficient, LAB-dominated fermentation process.

## Figures and Tables

**Table 1:** Effect of different additives on the fermentation quality of sheep grass silage.

	pH	LA	AA	PA	BA	NH_3_-N
		g/kg DM	g/kg DM	g/kg DM	g/kg DM	%FW
LP	4.26 ± 0.02 B	65.24 ± 0.42 A	25.81 ± 0.94 A	7.23 ± 0.34 B	0.00 ± 0.00 B	0.83 ± 0.00 A
LB	4.34 ± 0.05 B	49.59 ± 0.38 C	15.66 ± 0.55 BC	4.77 ± 0.38 C	0.11 ± 0.11 B	0.59 ± 0.13 A
CE	4.68 ± 0.03 A	54.64 ± 1.93 B	12.33 ± 1.21 C	11.89 ± 0.42 A	0.00 ± 0.00 B	0.63 ± 0.00 A
CK	4.72 ± 0.05 A	41.90 ± 1.34 D	16.04 ± 1.14 B	5.40 ± 0.29 C	1.72 ± 0.94 A	0.71 ± 0.00 A
*p*-value	0.0002	<0.0001	0.0004	<0.0001	0.0724	0.4972

Note: The value following the “±” sign represents the standard error of the mean (SEM). FW stands for Fresh Weight. DM stands for Dry Weight. LA lactic acid, AA acetic acid, PA propionic acid, BA butyric acid, NH_3_-N ammonia nitrogen, LP *L. plantarum*, LB *L*. *buchneri*, CE composite enzyme, CK no-addition control. Values with different uppercase letters (A–D) show significant differences among additives on the same ensiling day (*p* < 0.05).

**Table 2:** Effect of different additives on nutrient content of sheep grass silage.

	DM	WSC	CP	EE	ADF	NDF
	g/kgFW	g/kgDM	g/kgDM	g/kgDM	g/kgDM	g/kgDM
LP	581.62 ± 5.62 A	24.76 ± 0.18 B	70.94 ± 0.43 A	19.12 ± 0.09 B	438.53 ± 0.44 C	656.51 ± 2.73 B
LB	561.88 ± 9.69 AB	19.58 ± 0.31 C	63.98 ± 1.17 B	20.84 ± 0.33 A	450.74 ± 4.61 B	703.03 ± 2.71 A
CE	541.05 ± 7.13 B	24.02 ± 0.25 B	64.29 ± 0.32 B	18.37 ± 0.41 BC	428.81 ± 4.16 C	628.43 ± 4.08 C
CK	494.73 ± 6.44 B	12.81 ± 0.32 C	62.45 ± 0.46 B	17.49 ± 0.54 C	453.53 ± 3.53 B	698.74 ± 1.43 A
ORI	581.60 ± 5.57 A	46.44 ± 0.29 A	70.51 ± 0.94 A	20.72 ± 0.36 A	478.92 ± 3.40 A	704.82 ± 5.88 A
*p*-value	0.0002	<0.0001	0.0004	<0.0001	0.0724	0.4972

Note: The value following the “±” sign represents the standard error of the mean (SEM). FW stands for Fresh Weight. DM stands for Dry Weight. DM dry matter, CP crude protein, EE ether extract, WSC water-soluble carbohydrates, ADF acid detergent fiber, NDF neutral detergent fiber, LP *L*. *plantarum*, LB *L*. *buchneri*, CE composite enzyme, CK no-addition control, ORI native pasture sample for sheep grass. Values with different uppercase letters (A–C) show significant differences among additives on the same ensiling day (*p* < 0.05).

## Data Availability

The raw data supporting the conclusions of this article will be made available by the authors on request.

## Abbreviations

DM: Dry matter; CP: Crude protein; ADF: Acid detergent fiber; NDF: Neutral detergent fiber; WSC: Water soluble carbohydrates; LA: Lactic acid; AA: Acetic acid; PA: propionic acid, BA: butyric acid; NH_3_-N: Ammonia nitrogen; LP: *L. plantarum*; LB: *L. buchneri*; CE: composite enzyme.
